# Aggregation of Lipid-Anchored Full-Length H-Ras in Lipid Bilayers: Simulations with the MARTINI Force Field

**DOI:** 10.1371/journal.pone.0071018

**Published:** 2013-07-26

**Authors:** Hualin Li, Alemayehu A. Gorfe

**Affiliations:** Department of Integrative Biology and Pharmacology, University of Texas Medical School at Houston, Houston, Texas, United States of America; University of Leeds, United Kingdom

## Abstract

Lipid-anchored Ras oncoproteins assemble into transient, nano-sized substructures on the plasma membrane. These substructures, called nanoclusters, were proposed to be crucial for high-fidelity signal transmission in cells. However, the molecular basis of Ras nanoclustering is poorly understood. In this work, we used coarse-grained (CG) molecular dynamics simulations to investigate the molecular mechanism by which full-length H-ras proteins form nanoclusters in a model membrane. We chose two different conformations of H-ras that were proposed to represent the active and inactive state of the protein, and a domain-forming model bilayer made up of di16:0-PC (DPPC), di18:2-PC (DLiPC) and cholesterol. We found that, irrespective of the initial conformation, Ras molecules assembled into a single large aggregate. However, the two binding modes, which are characterized by the different orientation of the G-domain with respect to the membrane, differ in dynamics and organization during and after aggregation. Some of these differences involve regions of Ras that are important for effector/modulator binding, which may partly explain observed differences in the ability of active and inactive H-ras nanoclusters to recruit effectors. The simulations also revealed some limitations in the CG force field to study protein assembly in solution, which we discuss in the context of proposed potential avenues of improvement.

## Introduction

Ras proteins are membrane-associated enzymes that mediate a variety of signal transduction pathways [1], [2], [3], [4], [5]. They act as binary switches that are turned off when GDP bound and on when GTP bound. Exchange of GDP for GTP is facilitated by guanine nucleotide exchange factors [6], [7], [8], [9]. GTP loading leads to a conformational change on key regions of Ras that are required for interaction with effector proteins to transmit external signal to the nucleus. Signaling is turned off by GTP hydrolysis, which is catalyzed by G-protein activating proteins. In addition to these two well-characterized conformational states, recent studies have demonstrated that Ras can adopt intermediate substates that differ in affinity for effectors and exchange factors [10], [11]. Furthermore, a fraction of Ras proteins on the plasma membrane (PM) forms small, transient proteolipid assemblies (or nanoclusters). Nanoclusters serve as the exclusive sites of effector recruitment and activation [12], [13], [14]. The distribution of Ras nanoclusters on the heterogeneous surface of the PM varies with the activation status of the protein [13], [15]. These observations suggest that the biological activity of Ras involves additional, albeit poorly understood, checkpoints that are not fully captured by the commonly used on/off binary model.

The goal of the current work was to lay the foundation for a better understanding of the molecular basis for the predicted conformation-dependent clustering of Ras proteins [13]. Our focus is on H-Ras, one of the ubiquitously expressed human Ras isoforms whose structure and biochemistry is the best studied in the family [2]. H-Ras contains a conserved N-terminal catalytic G-domain (residues 1–166), and a hypervariable C-terminus comprising a linker (residues 167–179) and lipid-anchor (180–186) segments. Although the catalytic domain and the linker are able to modulate membrane binding [13], [16], [17], [18], [19], the farnesylated and dually palmitoylated lipid anchor (tH) is essential for the attachment of H-Ras to the inner leaflet of the PM [15], [20], [21], [22]. For instance, in addition to the lipid anchor, two basic amino acids on the G-domain of GTP-H-Ras were found to directly interact with the membrane [16], [17], [19]. In GDP-H-Ras another pair of basic residues from the linker help maintain a different membrane orientation of the catalytic domain [17], [19]. Subtle differences in these interactions have been shown to partially explain functional differences among Ras isoforms [16], [17].

An interesting question is how conformational change might lead to different nanoclustering and functional behavior of Ras proteins. Initial clues regarding this issue have been gleaned from recent experiments based on pressure perturbation coupled with a variety of spectroscopic techniques [23], [24], [25], [26], [27]. These efforts can be complemented by molecular simulation approaches. One powerful simulation technique that has the potential to yield atomically detailed information on Ras membrane binding and assembly is molecular dynamics (MD), particularly coarse-grained MD (CG-MD). CG-MD has been successfully used to study protein-lipid assemblies in large spatiotemporal scales [28], [29], [30], [31], [32], [33], [34], [35], [36], [37], [38], [39], [40], [41]. Applying CG-MD simulations on the minimal membrane-binding motif of H-Ras (tH), we have recently shown that 30–40% of these molecules spontaneously assemble into dynamic clusters of size 4–11 [42], [43]. The clusters localize at the boundary between liquid order (L_o_) and liquid disordered (L_d_) lipid domains [42], [43], [44]. Others have shown that full-length N- and H-Ras also form aggregates whose lipid domain preference is largely dictated by the lipid anchor palmitoyls [27], [45]. Here we ask if, and how, conformational change might modulate clustering and/or domain preference of full-length H-Ras. To this end, we carried out a series of long CG-MD simulations on much larger systems in which mixtures of lipids and the oncogenic G12V variant of H-Ras were allowed to self-assemble both in solution and in bilayer environments. We found that full-length H-Ras forms aggregates that exhibit conformation-dependent variabilities in terms of protein-protein and protein-lipid interactions. Moreover, analysis of the time evolution and equilibrium properties of the aggregates suggests that not only the lipid anchor but also the rest of the protein contributes to oligomerization. However, limitations associated with the CG model as well as the lack of additional membrane components led to aggregates that are larger and more stable than could expected from the literature data. We therefore used our simulations as a test of the CG model and suggested potential avenues for future improvement.

## Methods

### Model systems

**Figure 1:**
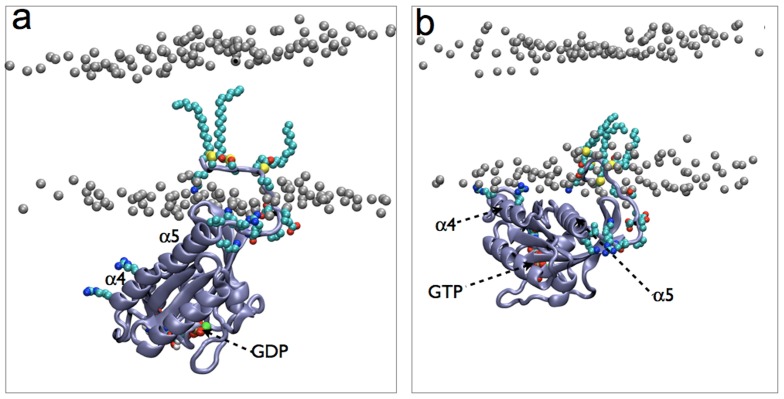
Two modes of membrane binding by H-Ras were observed in previous simulations [17]. (a) An approximately perpendicular orientation of the catalytic domain with respect to the membrane plane. This conformation, which we refer to as conf1, was frequently sampled during GDP-H-ras simulations [17]. (b) Semi-parallel orientation of the catalytic domain with respect to the membrane plane, which we refer to here as conf2. These two conformations were used as starting structures for the current simulations. Helices 4 and 5, as well as the bound nucleotide, are labeled. The canonical switches SI and SII are at the bottom of the image surrounding the nucleotide. Also shown in space-filling representation are side chains of the HVR along with Arg128 and Arg135 on helix 4, as well as the three lipid modifications (two palmitoyls at positions 181 and 184 and a farnesyl at position 186).

We simulated eight different systems using the MARTINI force field [29] (Table 1). The first two simulations (B1 and B2) involved a fully solvated bilayer containing 32 full-length H-Ras (Ras from here on) proteins in two different conformations. The major difference between the two conformations lies in the orientation of the catalytic domain with respect to the membrane plane (Figure 1). In one case the protein was approximately perpendicular to the membrane plane with the catalytic domain fully in water (conf1). In the second conformation helix 4 of the catalytic domain was roughly parallel to the membrane plane (conf2). Conf1 and conf2 represent the predominant conformations of GDP- and GTP-H-Ras proposed by atomistic MD simulations in a DMPC bilayer [17], and validated by cell biological and biophysical experiments [13], [16], [23], [24], [46]. Therefore, the G-domain and the linker were derived from the earlier simulations [17] and mapped into CG level and the lipid anchor was modeled as described before [42], [43], [44]. The proteins were embedded into the lower leaflet of a mixed lipid bilayer composed of 3480 di-16:0-PC (DPPC), 2304 di-18:2-PC (DLiPC) and 1536 cholesterol (CHOL) molecules (5:3:2 ratio), ensuring that each protein molecule is at least 5 nm away from its neighbors.

**Table 1:** Summary of the simulations.

Medium	Simulation name	Ras conformation	# of CG beads	Simulation length (μs)	Approx. Ras aggregation time (μs)
**Bilayer + sW**	B1	Conf1	267476	25	6
	B2	Conf2	203080	25	7
**sW**	W1	Conf1	187404	7	4
	W2	Conf2	149363	7	6
	W3	G-domain	174927	7	6
**pW**	pW1	Conf1	418361	7	4
	pW2	Conf2	418361	7	4
	pW3	G-domain	487442	7	6

sW and pW stand for the standard and polarized water models of MARTINI. Full-length H-ras was simulated in two different conformations (conf1 and conf2) whereas only a single conformation of the catalytic G-domain was used.

A second set of three simulations was carried out in solution in order to examine the role of membrane binding for oligomerization. Two of these simulations (W1 and W2) contained the full-length H-Ras in conformation in conf1 and conf2, respectively, while the third (W3) contained the isolated catalytic domain (i.e., without the hypervariable region). The latter was included to assess the importance of the hypervariable region for aggregation. Since all five of these simulations used the standard MARTINI water model (sW) [28], the potential impact of the solvent model was evaluated by repeating the latter three simulations with the latest polar water model of MARTINI [47] (simulations pW1, pW2 and pW3).

### Simulation details

For all simulations the box dimension was 44 nm×46 nm in length and width but vary in the z dimension from 14 nm (B1) to ∼10 nm (B2-pW3), depending on the presence or absence of a bilayer and whether the isolated G-domain or the full-length protein was considered. Appropriate numbers of water molecules plus sodium and chloride ions (ionic strength = 0.05–0.06 M) were added to each system (see Table 1).

All simulations were performed with the GROMACS package version 4.5.3 [48] using an integration time step of 20 fs, the neighbor list for pairwise non-bonded interactions was determined by a cutoff of 1.35 nm and updated every 10 steps. Other standard MARTINI prescriptions used here include shifting the Coulomb interactions to zero between 0 and 1.2 nm and the van der Waals interactions between 0.9 and 1.2 nm, as well as the use of periodic boundary conditions to account for finite size effects. An isothermal-isobaric ensemble (NPT) was used at 1 bar and 301 K. Constant pressure were maintained by a semi-isotropic, weak coupling scheme using a relaxation time of 2ps. Constant temperature was maintained by separately coupling protein plus lipids and water plus ions to a 301 K heat bath using a relaxation time τ_T_  =  1 ps. An effective dielectric constant of 15 and 2.5 was used in simulations with the standard and polarizable MARTINI water models, respectively. After energy minimization and equilibration, the bilayer systems were run for 25 μs and those without bilayer for 7 μs (i.e., 100 μs and 28 μs effective time, respectively [28]). Elastic networks were applied to preserve higher-order protein structures in all simulations [49] and, in the case of B2, membrane orientation of the G-domain was maintained by applying additional distance restraints on selected backbone beads of the anchor and the linker. The restraints were based on inter-atomic distances in the initial structure of conf2 (see Table S1), and their effect on the protein structure is illustrated in Figure S1.

### Data analysis

VMD [50], GROMACS [48] and local tools were used for visualization and data analysis. Each trajectory was divided into an initial phase in which lipid segregation and/or protein aggregation was incomplete, and an equilibrated phase in which segregation/aggregation was complete. The first was used to evaluate the progress of Ras clustering and the latter to characterize the equilibrium properties of the aggregates. Two proteins were considered to be in contact (or interacting) when any two of their backbone beads are within 0.75 nm of each other. Similarly, proteins are assumed to be in the same cluster if any backbone bead of one protein is within 0.75 nm of that of another. The lateral self-diffusion coefficient, D, of proteins and lipids was calculated from a linear regression of the time dependent mean square displacement (MSD) based on Einstein relation [51]:
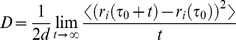
(1)where *d = 2* is the dimensionality of the system, r_i_ is the displacement , τ_0_ and t stand for the initial time and the specific time duration.

To investigate protein-protein interactions during and after Ras aggregation, we used two residue-based measures: contact probability per residue, *P*, and the change in residue solvent accessible surface area (ΔSASAs). *P* was calculated as:
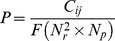
(2)


where *C_ij_* is the number of contacts between residue *i* in one molecule and residue *j* in another, *F* is the number of frames, 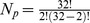 is the total possible outcome of randomly choosing 2 protein molecules out of 32 that exist in the system, and *N_r_* is the number of residues per protein molecule. *N_r_* is *186* for full-length Ras and 166 for the isolated domain. As mentioned above, *C_ij_* was determined based on the criterion that two residues in different proteins are in contact if their backbone beads are within 0.75 nm [42], and the normalization term 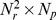 stands for the contact probability between a random residue in one molecule and another random residue in a different molecule. Solvent accessible surface area (SASA) differences between the final aggregated state and the initial monomeric state (or ΔSASA) should capture regions of the protein surface that become buried at protein-protein interfaces. In other words, since residues with large negative ΔSASA (i.e. those accessible to solvent before aggregation but not after) are expected to take part in protein-protein interactions, ΔSASA represents the buried surface area of a residue upon protein aggregation. For example in simulations B1 or B2, the difference in SASA of a residue at the end (SASA_25 µs_) and the beginning (SASA_0 µs_) of the simulation is (ΔSASA = SASA_25 µs_–SASA_0 µs_). SASA was calculated using a probe radius of 0.56 nm, which was about four times the typical value used in atomistic models (0.14 nm). As noted previously [52], this was required to account for the larger bead radius that maps four heavy atoms.

For visualization purposes, CG structures were mapped back to atomistic models using a modified version of the CG2AT mapping procedure described by the Sansom group [53]. Briefly, our procedure involves using PULCHRA [54] to reconstruct backbone heavy atoms from the corresponding CG beads, followed by applying MODELLER [55] and its “complete_pdb()” function to add missing atoms. The resulting model was optimized by energy minimization. Structural and compositional features of the bilayer were analyzed as described previously [42]. The effect of Ras aggregates on the mechanical property of the host bilayer will be discussed elsewhere.

## Results and Discussion

We first describe how full-length Ras forms aggregates in a bilayer of coexisting striped lipid domains. We then examine how variation in the initial conformation of the protein modulates protein-protein interaction and membrane organization of the final aggregate. We then discuss the limitations of our model systems and potential avenues for improvement.

### Insertion and aggregation of full-length H-ras in a lipid bilayer

**Figure 2:**
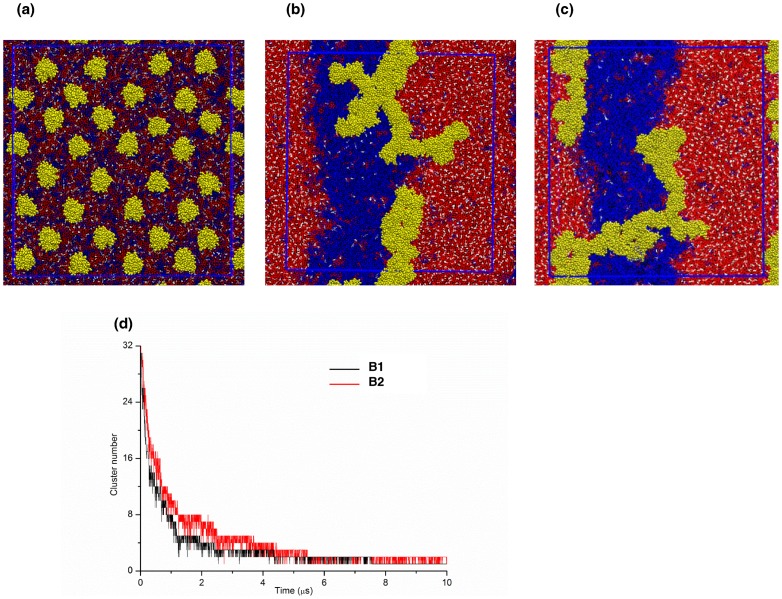
Snapshots and aggregation profiles derived from simulations B1 and B2. Top view of initial (a) and final configurations of conf1 (b), and conf2 (c), in a 5:3:2 DPPC:DLiPC:CHOL ternary bilayer. The 32 H-ras proteins are colored in yellow, DPPC in red, DLiPC and CHOL in blue and white. (d) The number of total clusters during the simulations.

Figure 2a shows the top view of an initial setup of one of the bilayer simulations in which 32 Ras molecules were evenly distributing at the lower leaflet. The final snapshots show two striped lipid domains (Figure 2b and 2c): a smaller domain dominated by the unsaturated DLiPC lipids and another largely devoid of DLiPC but enriched with DPPC and cholesterol. Lipid de-mixing was complete in less than 10 µs in each simulation (Figure S2). Consistent with results from numerous previous simulations [32], [36], [42], [43], [44], the DLiPC- and DPPC/cholesterol-enriched domains exhibit features (such as thickness) typical of an L_d_ and L_o_ domain, respectively.

Atomistic [56], [57], [58], [59] and CG [42], [43], [45] simulations as well as solid-state NMR experiments [60], [61], [62], [63], [64], [65] have shown that protein-lipid interactions via the lipidated motif of Ras is necessary and sufficient for membrane binding. Therefore, our simulations were set up in such a way that the hydrophobic palmitoyl and farnesyl tails of each Ras molecule were inserted deep into the lower leaflet of the lipid bilayer. In the course of the simulations, the Ras lipids remained inserted into the hydrophobic core while the backbone of the lipid anchor remained at the hydrophobic/hydrophilic interface. Apart from some variations in the insertion depth and specific protein-lipid contacts none of the 32 protein molecules dissociated during the simulations.

Figure 2b and 2c show that, irrespective of the initial conformation, a single, large aggregate was formed at the end of the simulations. As in tH [42], aggregation did not seem to require lipid domain formation. This can be seen from Figure 2d, where we plotted the time evolution of the total number of Ras clusters. Aggregation was complete within about 4–6 μs in both B1 and B2, which is faster than the time it took for complete lipid de-mixing (6–8 μs) (Figure S2). The decay rates in Figure 2d also indicate that aggregation kinetics was only marginally affected by protein conformation, with the speed of aggregation in B2 being slightly slower perhaps due to the much larger contact surface of conf2 with the bilayer (see Figure S3). As already mentioned, monomers and smaller clusters disappeared within about 6 µs in both B1 and B2, giving way to a single, large and semi-linear assembly. This is in contrast to earlier experiments [12], [13], as well as the aggregation behavior of tH observed in previous simulations [42]. At any given time of the previous simulations, only a fraction of tH molecules (∼40%) exist in multiple, dynamic clusters of average size ∼6 [42]. The reasons for the uncontrolled cluster growth in the current simulations could be manifold, and likely include limitation in the CG model and the simplicity of our model membrane. For example, coarse-graining does not allow for accurate representation of certain polar interactions, such as hydrogen bonds or salt bridges, and the use of restraints to preserve protein secondary structures limits internal dynamics. These and other limitations of our model are discussed in another section. Here we focus on general observations that are less sensitive to the level of detail/complexity of the model, such as lateral dynamics and large-scale reorganization during aggregation, and how these are modulated by conformational change. The potential implications of our findings for Ras function are discussed only sparingly.

### Protein-protein interactions during and after H-Ras aggregation

**Figure 3:**
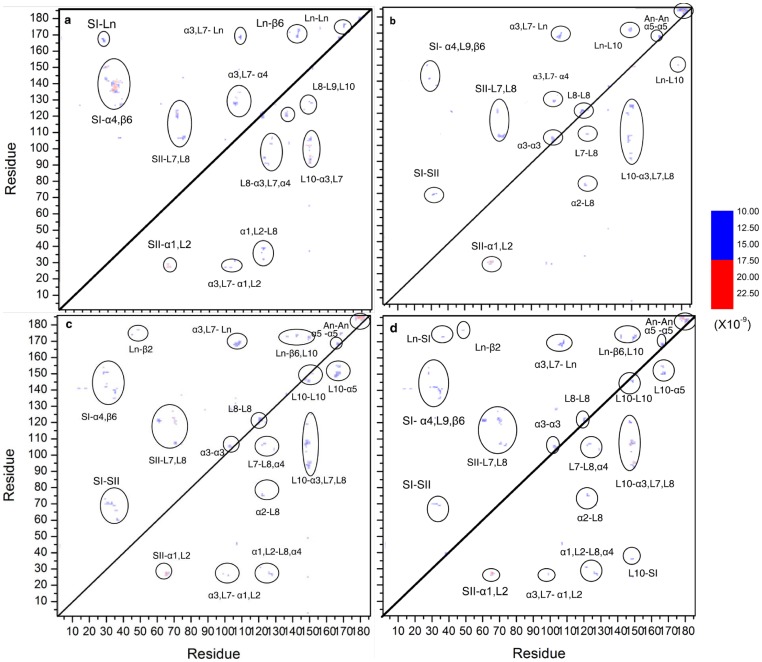
Protein-protein contact probability map for conf1 (upper half) and conf2 (lower half) during different stages of the simulations: (a) 1–2 µs, (b) 9–10 µs, (c) 19–20 µs, and (d) 24–25 µs. The color scale on the right refers to the *P* value (the same scale is in Figures 4b and S4). All *P* values were calculated from the number of residue-residue contacts normalized as indicated in eqn2. The critical contact regions are highlighted with circles and labeled by Ln: linker, An: anchor, L: loop, SI: switch I, SII: switch II, α: α helix, β: β-strand.

Despite the limitations mentioned above, our simulations captured some of the basic characteristics of Ras clustering that would be impossible to see in atomistic simulations. These include protein-protein interactions that drive aggregation, which we have quantified by pairwise residue contacts (*P*, see eqn 2). We calculated *P* at different time windows of simulations B1 and B2 to compare changes in inter-protein contacts at the nucleation, progression and stabilization phases of Ras aggregation. Figure 3 shows that the majority of the initial contacts involved semi-random contacts across the protein surface. Cluster growth was accompanied by significant conformational reorganization involving the appearance of new contacts and disappearance of old ones. In particular, contacts in the hypervariable C-terminus became more frequent as aggregates grew, though reduced to some extent later on as contacts at the G-domain increased. Nonetheless, the contact pattern remained unchanged after the formation of a single large aggregate.

**Figure 4:**
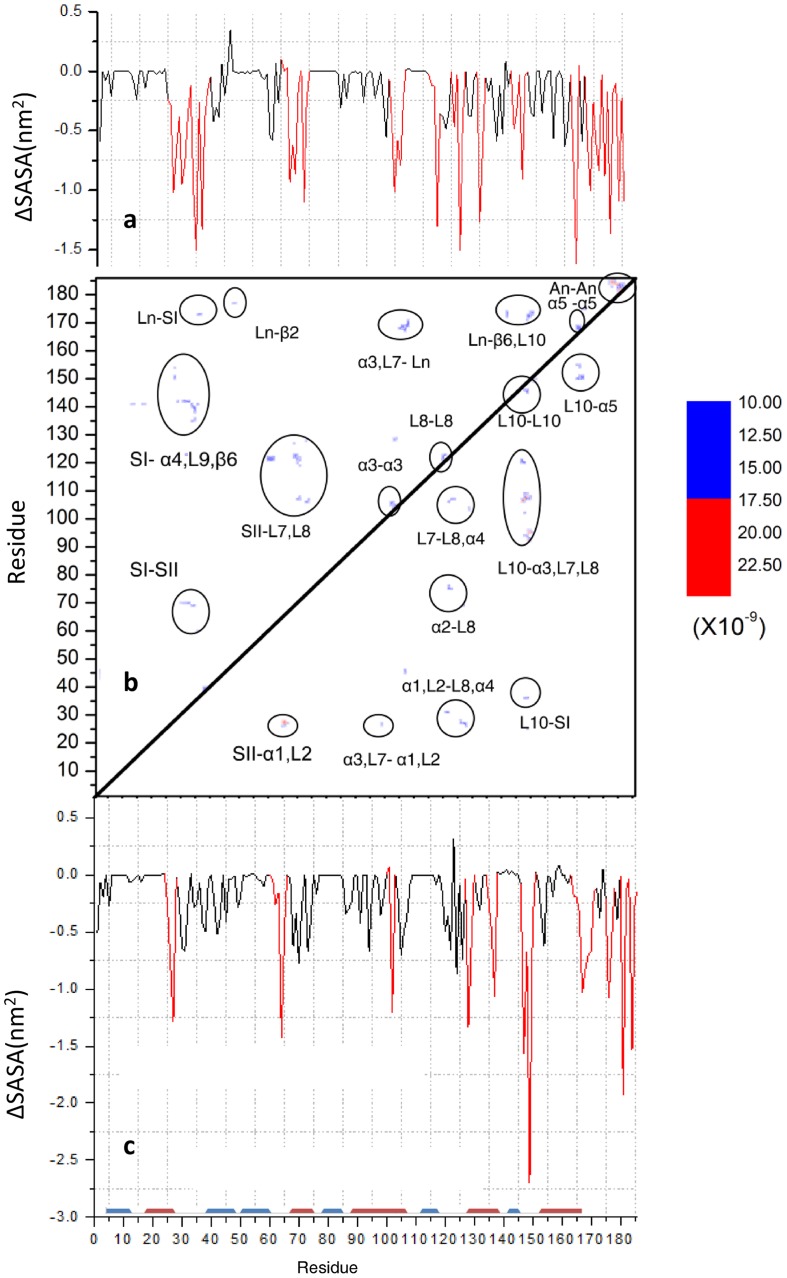
Contour maps of P for the snapshots sampled from the 24–25 µs window, with key regions highlighted as in Figure 3 (upper half: conf.1, lower half: conf2). The corresponding ΔSASA distributions are displayed next to the contour maps. Residues with ΔSASA>0.75 nm^2^ are highlighted in red. Average SASA was calculated on 4ns-spearated frames and averaged over the last and first 1 µs windows. Major secondary structure are indicated schematically with helices in red and strands in blue in (c).

**Figure 5:**
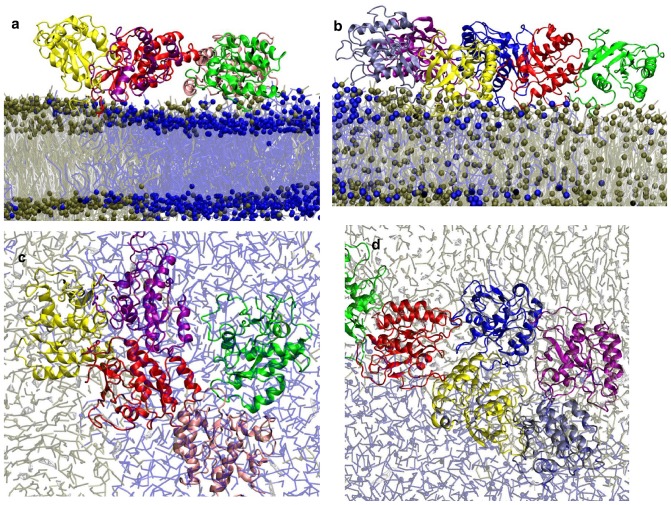
Snapshots illustrating inter-protein interactions in Ras aggregates derived from simulations with Ras in conf1 (left) and conf2 (right). Shown are side (a & b) and top views of conf1 and conf2 aggregates. The color scheme for the lipid molecules is the same as in Figure 1 (except for DPPC, which is in now tan). Proteins are shown in different colors.

Figure 3d compares the intermolecular contacts in the final aggregates of conf1 and conf2. The contact patterns within conf1 and conf2 aggregates significantly differ, especially at residues with high contact probability (arbitrarily defined here as those with P>10.0×10^−9^, see Tables S2 and S3). Interestingly, many of the differential contacts involve regions of Ras that are important for effector/modulator binding, including the conformationally responsive switch I (SI, residue 30–40) and switch II (SII, residues 57–75), as well other surface loops (Figures 3 and 4). Both SI and SII are involved in inter-protein interactions in the aggregates, but they do so more prominently in conf1 than in conf2 (see Tables S2 and S3 for details). Snapshots illustrating inter-protein interactions in Ras aggregates in the final stage of simulations are shown in Figure 5. For visualization purposes, CG structures of Ras were mapped back to atomistic model as described previously.

To further explore the protein-protein interfaces within the aggregates, we compared changes in residue solvent accessibility (ΔSASA) in simulations B1 and B2 (Figure 4a and 4c). One can see that for both conf1 and conf2, residues with high *P* scores (i.e., residues frequently involved in intermolecular contact) also exhibit large ΔSASA, which is roughly defined as those with ΔSASA >0.75 nm^2^ and highlighted in Figure 4a and 4c with red. The conf1 and conf2 aggregates share few overlapping regions that have elevated values of both P and ΔSASA. In conf1, the regions with elevated P and ΔSASA include SI and SII. In conf2 the HVR and helix 4 have high P and ΔSASA. It is clear that a number of residues in each of these regions were accessible to solvent before aggregation but became almost completely buried after aggregation. This provides additional evidence for their interfacial nature and role in conformation dependent formation and/or stability of H-Ras aggregates. In addition, part of helix 5 contributes to contact formation in conf2 but less in conf1. This suggests that in the latter, interactions via helix 5 compensate for the lack of extensive contact around the switch regions that exist in conf1. It is worth noting that a recent study of N-Ras in a POPC bilayer predicted an important role for helix 5 residues in dimer formation [25].

Analyses of P and ΔSASA identify the lipid anchor as being critical for the formation and/or stability of conf1 and conf2 aggregates (Figure 4), which is consistent with the documented role of the Ras lipid tails for cluster formation by tH [43], [44]. To further evaluate the significance of the lipid anchor for clustering of full-length Ras, we looked at protein-protein contacts in conf1/conf2 aggregates in the absence of bilayer (i.e., simulations W1 and W2). The idea was that the hydrophobic interaction among the apolar lipid anchors would be enhanced in the more polar solvent environment relative to the bilayer core. In other words, if interactions between residues at the catalytic domain are dominant, then simulations with and without bilayer will give rise to the similar contact interface. The data in Figure S4a and S4b show that the protein-protein interfaces have largely shifted from the catalytic domain to the lipid anchor in simulations W1 and W2. Thus, the lipid anchor provides the dominant driving force for the oligomerization of H-ras in solution. This effect is tempered by the bilayer because the reduced dimensionality limits the orientational freedom of the protein and thereby reduces the ability of neighboring lipid anchors to form close contacts.

### Lateral dynamics and distribution of H-Ras oligomers

**Figure 6:**
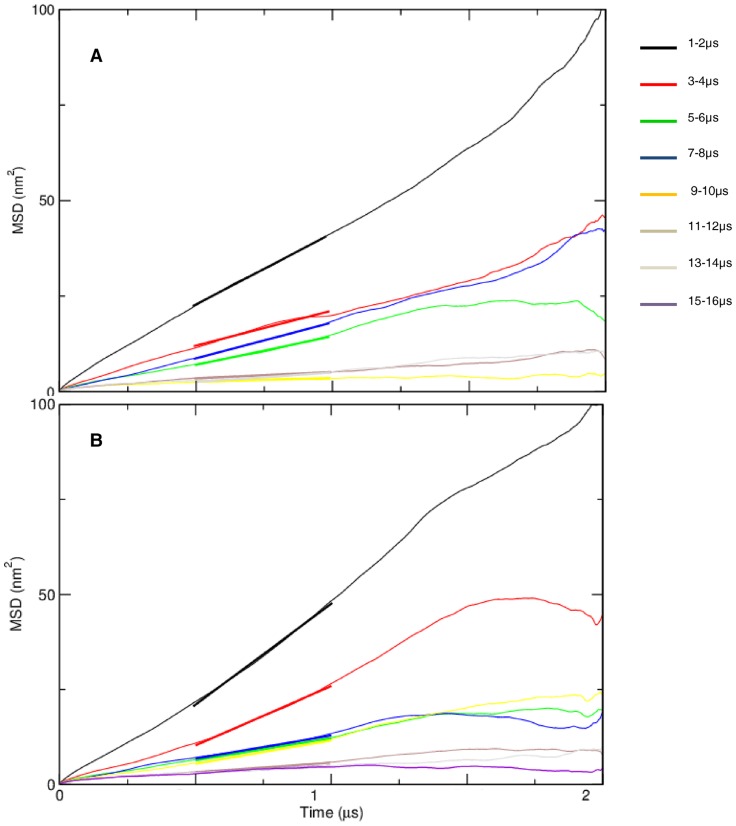
Mean square displacements of conf1 (a) and conf2 (b) during the indicated time windows. The lateral diffusion coefficient was calculated from a linear fit to the MSD curve in the time interval highlighted by bold lines.

The lateral displacement of H-Ras was monitored by the self-diffusion coefficient (D) calculated during and after aggregation (see Figure 6). The results were compared with the diffusion coefficients of each lipid type before and after phase separation (Table 2 and Figure S5). We found that Ras monomers in conf1 diffuse at approximately the same rate as DLiPC lipids before phase separation, whereas in conf2 Ras diffuses slightly slower and at roughly the same rate as DPPC lipids. After phase separation, D for DPPC and cholesterol decreased by ∼20% while that of DLiPC molecules increased by roughly the same magnitude. The final values are consistent with previous reports [32]. By contrast, for both conformations of Ras, D was reduced by ∼10 fold during aggregation and by an additional ∼5 fold after aggregation. This is expected because the increased size upon aggregation can reduce diffusion. As a result, dimers, trimers and larger aggregates have progressively slower rates of lateral displacement than monomers. The slightly slower diffusion of monomeric H-Ras in conf2 (Figure S6) can be understood from the additional contact the G-domain makes with the bilayer. To quantify this, we calculated the number of lipid beads in contact with the H-ras G-domain and linker regions (residue 1–179) for conf1 and conf2 (Figure S3). It can be seen that contact to the membrane by conf2 is ∼3 times more abundant than that of conf1. However, it is interesting to see that the contact surface of conf2 decreased by ∼17% during aggregation. This may be partly explain why the difference in D between conf1 and conf2 aggregates became negligible after complete aggregation. Another possible reason for the similar lateral dynamics of conf1 and conf2 aggregates might be due to the large size of the clusters, which greatly diminishes lateral movement in both cases.

**Table 2:** Lateral diffusion coefficient (D) of molecules in the bilayer (×10^−8^ cm^2^/s).

Molecule	DPPC		DLiPC		CHOL		H-Ras		
**Time (μs)**	1–6	7–16	1–6	7–16	1–6	7–16	0.04–0.16	3–8	9–14
**B1**	6.3	4.9	9.8	11.2	7.5	5.6	10.5	1.1	0.2
**B2**	6.8	4.5	9.0	10.5	5.0	5.2	6.8	0.8	0.3

D for H-Ras before, during and after aggregation was calculated for time windows 40–160 ns, 3–8 µs (5–10 µs in the case of conf2) and 9–14 µs (11–16 µs in the case of conf2). Errors estimated from block averaging range between 0.0 and 1.8, the larger errors being for the early stages of the simulations.

Visual inspection of the aggregates (Figure 2b and 2c) suggests that about half of the molecules in the aggregates are located at the boundary between the L_o_ and L_d_ domains. To quantify this observation, we calculated the number of L_o_ and L_d_ lipid beads that are within 0.75nm of conf1 and conf2 beads in the last one µs of the simulations. The ratio of L_o_ beads (DPPC) to L_d_ beads (DLiPC) in contact with proteins was found to be 0.85±0.01 and 1.21±0.02 for conf1 and conf2 aggregates, respectively. This indicates a preference for the boundary between the L_o_ and L_d_ domains, which is consistent with previous results on tH [42] and is partly a result of the competition between the saturated palmityol and the unsaturated farnesyl tails for interaction with L_o_ and L_d_ domains [42], [43], respectively.

### Possible limitations of the model, and future direction

Because CG simulations suffer from the necessary tradeoff between computational cost and interaction detail, the results should be interpreted with care. A case in point is that all 32 H-ras proteins formed a single, long and very stable linear cluster in the simulations, whereas in cells only a fraction of Ras molecules exist in multiple, small clusters [12], [13]. Among a number of possible reasons for this discrepancy, the following might be the most important. First, the MARTINI force field requires the application of restraints to preserve protein secondary structures. The resultant diminution of internal fluctuations could affect cluster size and dynamics. Secondly, there may be a strong attraction between a few pair of residues at the solvated catalytic domain, an issue discussed by the developers of MARTINI in various contexts [45], [66]. For instance, in their simulation of lipid-anchored proteins (including N-Ras), de Jong et al [45] observed the formation of linear aggregates that are very stable once formed. We note in this context that the ability of MARTINI to describe atomic interactions in water has not been extensively tested. The third potential limitation involves the water model. It was observed that “a collective effect that arises from too large de-wetting surfaces of MARTINI water beads” might lead to stronger interaction between proteins [45]. The polar MARTINI water model (pW) partially alleviates this problem [66], [67]. In our simulations, too, pW somewhat reduced the number of residue-residue contacts in the polar regions of the catalytic domain without significantly affecting the apolar interactions at the lipid anchor (Figure S4). To further examine the effect of pW, we compared the aggregation behavior of both the full-length Ras and the truncated catalytic domain in the standard (sW) and pW water models. To facilitate comparison, we calculated the ratio *R* between the total pairwise residue contacts (H) in the pW and sW simulations:
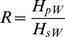
(3)


We found R values of 0.59, 0.66 and 0.83 for the isolated G-domain, full-length conf1 and full-length conf2, respectively. This clearly shows that the probability of protein-protein contact formation significantly decreases in the polar water, thereby reducing (but not eliminating) the tendency to form large aggregates. The improvement was more dramatic for the isolated catalytic domain, probably because the proportion of polar interactions (versus total) in the isolated catalytic domain is larger than that in the full-length protein. This is consistent with previous reports [45], [66], [67], and suggests that the polar water model improves inter-molecular interactions. Note that the current comparison was done in solvent (i.e., in the absence of bilayer). It is therefore possible that the use of pW in the context of a bilayer may prevent formation of a single aggregate. It would be interesting to see how significant this improvement would be. Finally, elastic networks used to maintain higher order structures (see SI) [29], [37], [45], [49], [68] may also affect protein-protein interaction. Furthermore, we applied additional restraints at the HVR of conf2 to maintain its orientation with the membrane (see SI), which may affect lateral dynamics somewhat.

We also note that our model membrane is simple compared with the rather complex plasma membrane with which the available experiments on H-Ras nanoclustering were carried out [12], [16]. For instance, the actin cytoskeleton has been shown to be required for the clustering of inactive H-Ras [12], and trans-membrane proteins may affect lateral diffusion by compartmentalizing smaller clusters.

Taken together, it is clear that there is much room for improving the predictive power of the simulations. Future simulation efforts aimed at mitigating the limitations of the CG model and incorporating additional membrane components that limit lateral diffusion and cluster growth may yield cluster sizes that better match experiments. This can be achieved, for instance, by incorporating obstacles (such as immobilized particles) into the bilayer [69].

## Conclusions and Implications for Ras Signaling

We performed extensive CG-MD simulations to examine cluster formation by full-length H-ras in two different conformations. Although simulations of H-ras in conf1 and conf2 led to single semi-linear assemblies, perhaps due to the intrinsic limitation of CG models to study the assembly of surface bound proteins, the protein-protein interactions during and after Ras aggregation showed significant differences. Intriguingly some of the key differences between the two models of H-Ras aggregates involved functional regions. Regions of the protein that are involved in protein-protein interaction will not be accessible to solvent after clustering, and therefore will become unavailable for interaction with other proteins. Assuming that aggregates of conf1 and conf2 represent clusters of inactive and active H-Ras, we speculate that differential accessibility of key regions of Ras to solvent will have implications for biological activity. In particular, the inaccessibility of the switch regions in conf1 (due to their involvement in protein-protein interaction) and their availability for interaction with other proteins in conf2, is consistent with activation state dependent Ras nanoclustering [13], and may explain the selective recruitment of Raf by active Ras nanoclusters [13], [16].

## Supporting Information

Figure S1 **Conf1 and conf2 bound to the bilayer.** (a) & (b) The role of elastic network applied on conf1 (a) and conf2 (b) were tested by comparing their G-domain orientation and secondary structure using snapshots at 0 μs (green) and 25 μs (red). The proteins are aligned based on the backbone beads of the G-domain and are illustrated by bonded snapshots at 0 μs (grey) and 25 μs (blue). For conf2, the vertical distance between the center of mass (COM) of helix 4 and the COM of all PO4 groups in the lipid molecules that are within 40 Å is defined as H. (c) Without the application of elastic networks, the tertiary structure fell apart within 1 μs. (d) Without the additional restraints in conf2 helix4 goes away from the bilayer, indicating that the restraints are necessary to keep conf2 properly oriented on the bilayer surface. The error bars were calculated from standard deviation of H for 32 Ras molecules.
(TIF)

Figure S2 **Time evolution of contact ratio between DPPC and DLiPC in the upper and lower leaflets in simulations B1 (a) and B2 (b) calculated as described before (Janosi L et al. (2012)** ***PNAS*** **109: 8097–8102.).** Lipid de-mixing was complete within 6–8 μs in both cases.
(TIF)

Figure S3 **Time evolution of the number of lipid beads that are in contact with Ras residues 1–179.**
(TIF)

Figure S4 **Contact probability P illustrated by contour maps averaged over the last microsecond of the simulations.** (a) W1 (upper)/W2 (lower), (b) pW1 (upper)/pW2 (lower), (c) W3 (upper)/pW3 (lower). Abbreviations are the same as in Figure 3.
(TIF)

Figure S5 **Mean square displacement of lipids for simulations B1 (left) and B2 (right).** (a) DPPC, (b) CHOL, (c) DLiPC. The lateral diffusion coefficient was calculated from a linear fit to the portion of the MSD curve highlighted in bold lines and shown in Table 2.
(TIF)

Figure S6 **Mean square displacement of H-ras monomers for simulation B1 (a) and B2 (b).** The lateral diffusion coefficient was calculated from a linear fit to the portion of the MSD curve highlighted in bold lines and shown in Table 2.
(TIF)

Table S1 **Additional distance restraints applied in conf2 to keep Ras orientation.**
(PDF)

Table S2 **Residue pairs for conf1 with high P values (cutoff = 10.0×10** ^−9^ **) over the last two microseconds of the simulations, with residue numbers and single letter codes of the amino acids listed in separate columns.**
(PDF)

Table S3 **Residue pairs for conf2 with high P values (cutoff = 10.0×10** ^−9^ **) over the last two microseconds of the simulations, with residue numbers and single letter codes of the amino acids listed in separate columns.**
(PDF)
