# Supplementary figures and images for: Aggregation of Lipid-Anchored Full-Length H-Ras in Lipid Bilayers: Simulations with the MARTINI Force Field
Source: PLoS One. 2013 Jul 26;8(7):e71018. doi: 10.1371/journal.pone.0071018 (PMC3724741; doi:10.1371/journal.pone.0071018)

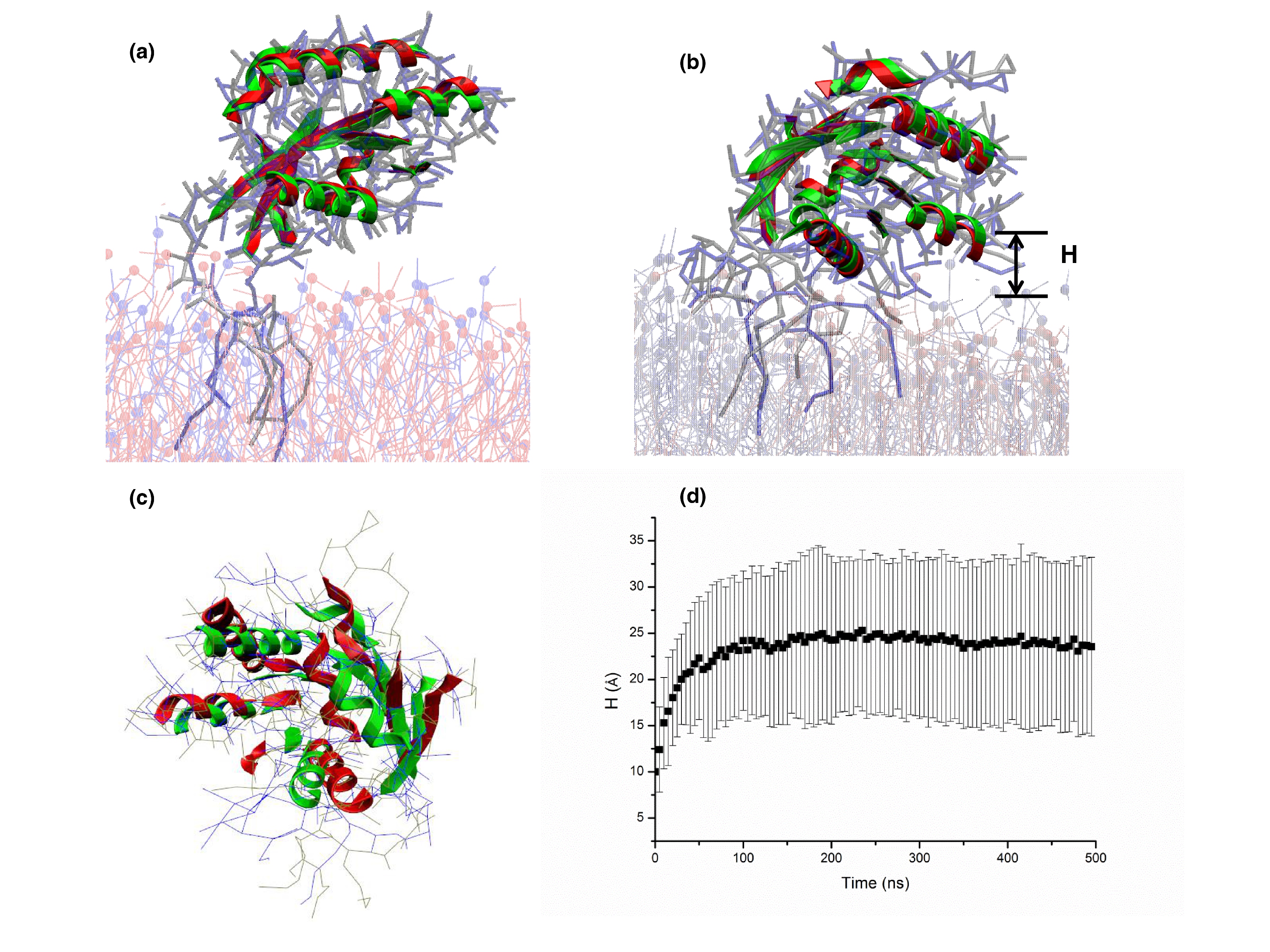

Supplement: Figure S1 — Conf1 and conf2 bound to the bilayer. (a) & (b) The role of elastic network applied on conf1 (a) and conf2 (b) were tested by comparing their G-domain orientation and secondary structure using snapshots at 0 μs (green) and 25 μs (red). The proteins are aligned based on the backbone beads of the G-domain and are illustrated by bonded snapshots at 0 μs (grey) and 25 μs (blue). For conf2, the vertical distance between the center of mass (COM) of helix 4 and the COM of all PO4 groups in the lipid molecules that are within 40 Å is defined as H. (c) Without the application of elastic networks, the tertiary structure fell apart within 1 μs. (d) Without the additional restraints in conf2 helix4 goes away from the bilayer, indicating that the restraints are necessary to keep conf2 properly oriented on the bilayer surface. The error bars were calculated from standard deviation of H for 32 Ras molecules. (TIF) [file pone.0071018.s001.tif]

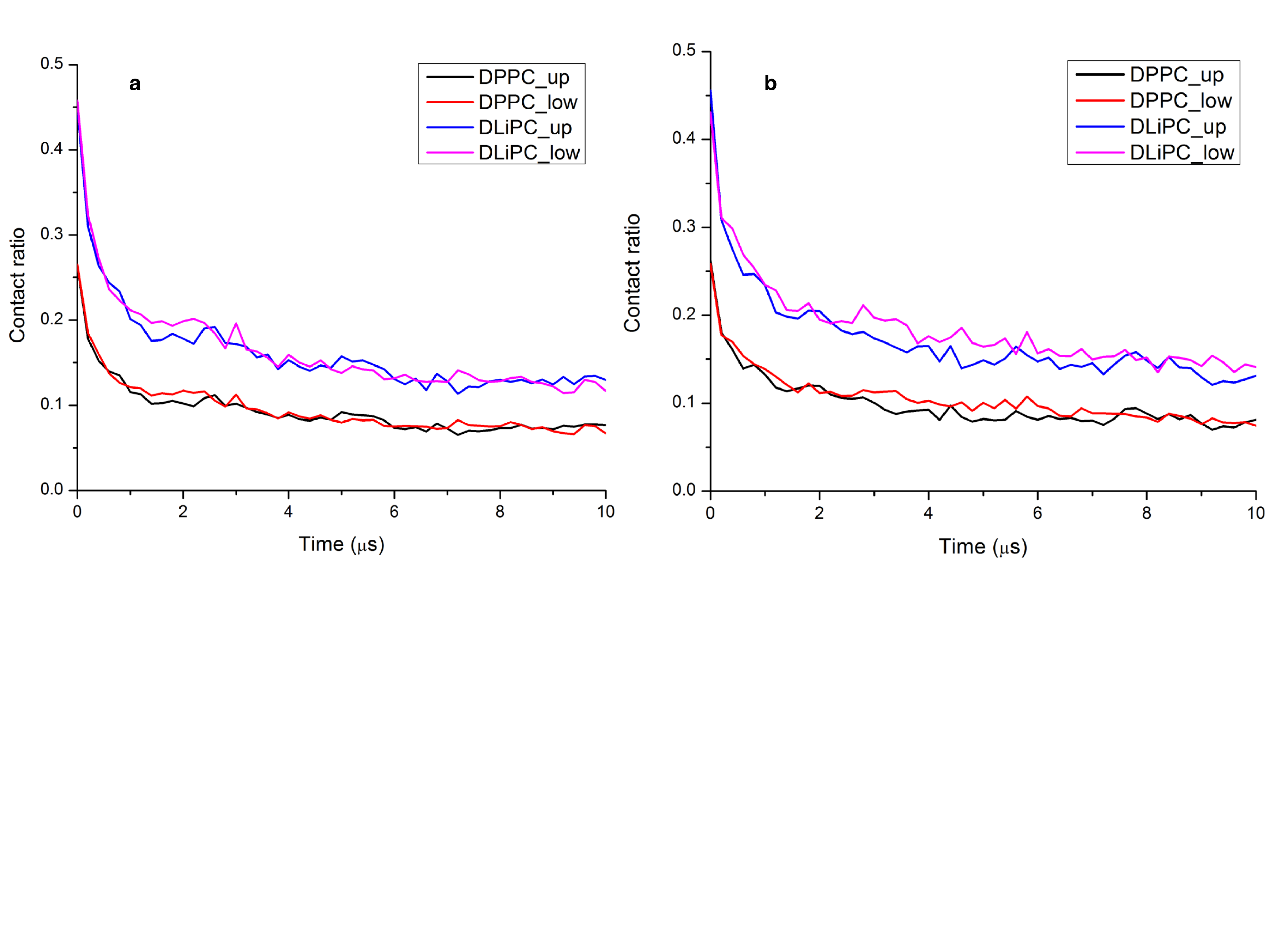

Supplement: Figure S2 — Time evolution of contact ratio between DPPC and DLiPC in the upper and lower leaflets in simulations B1 (a) and B2 (b) calculated as described before (Janosi L et al. (2012) PNAS 109: 8097–8102.). Lipid de-mixing was complete within 6–8 μs in both cases. (TIF) [file pone.0071018.s002.tif]

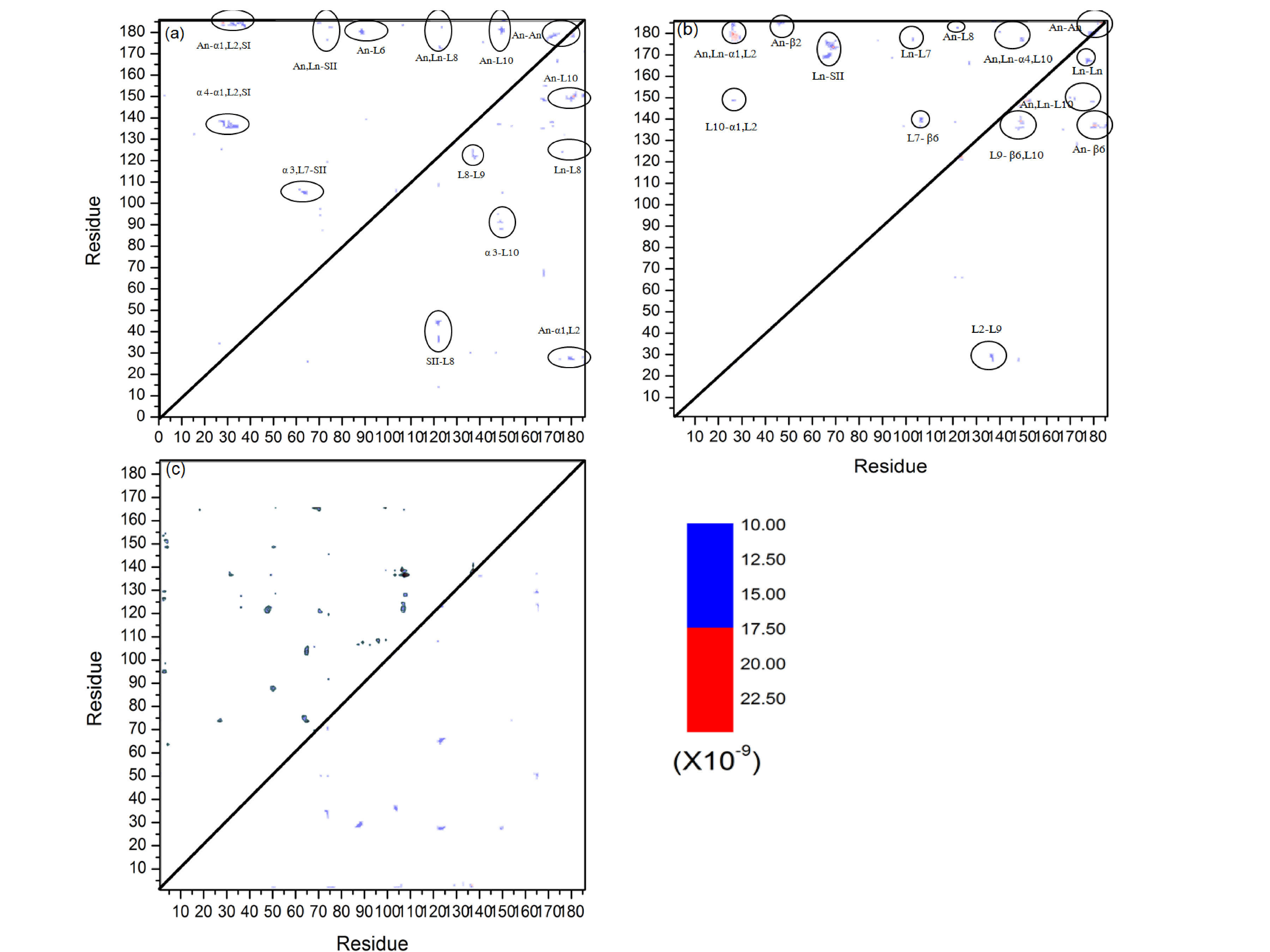

Supplement: Figure S4 — Contact probability P illustrated by contour maps averaged over the last microsecond of the simulations. (a) W1 (upper)/W2 (lower), (b) pW1 (upper)/pW2 (lower), (c) W3 (upper)/pW3 (lower). Abbreviations are the same as in Figure 3. (TIF) [file pone.0071018.s004.tif]

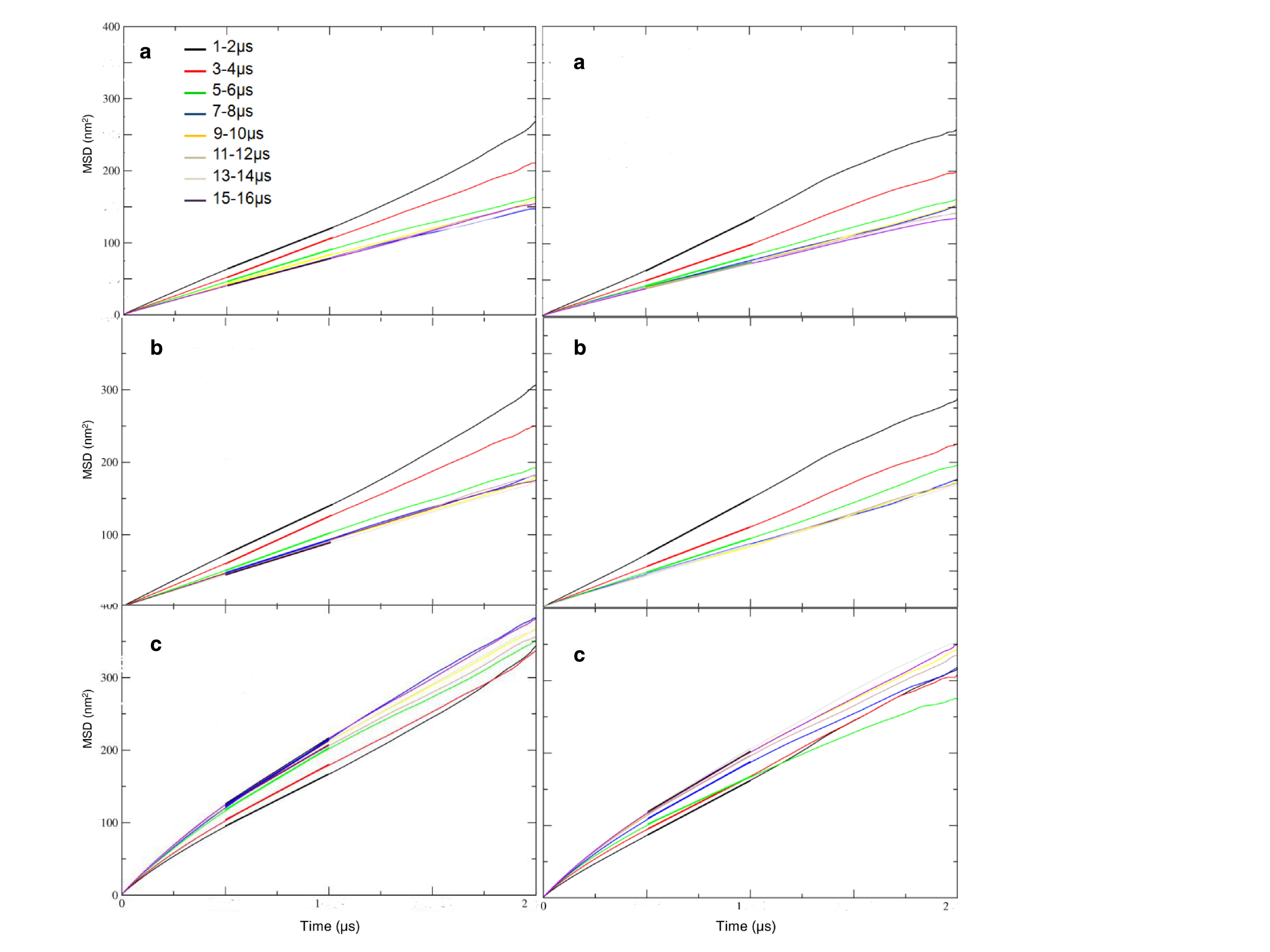

Supplement: Figure S5 — Mean square displacement of lipids for simulations B1 (left) and B2 (right). (a) DPPC, (b) CHOL, (c) DLiPC. The lateral diffusion coefficient was calculated from a linear fit to the portion of the MSD curve highlighted in bold lines and shown in Table 2. (TIF) [file pone.0071018.s005.tif]

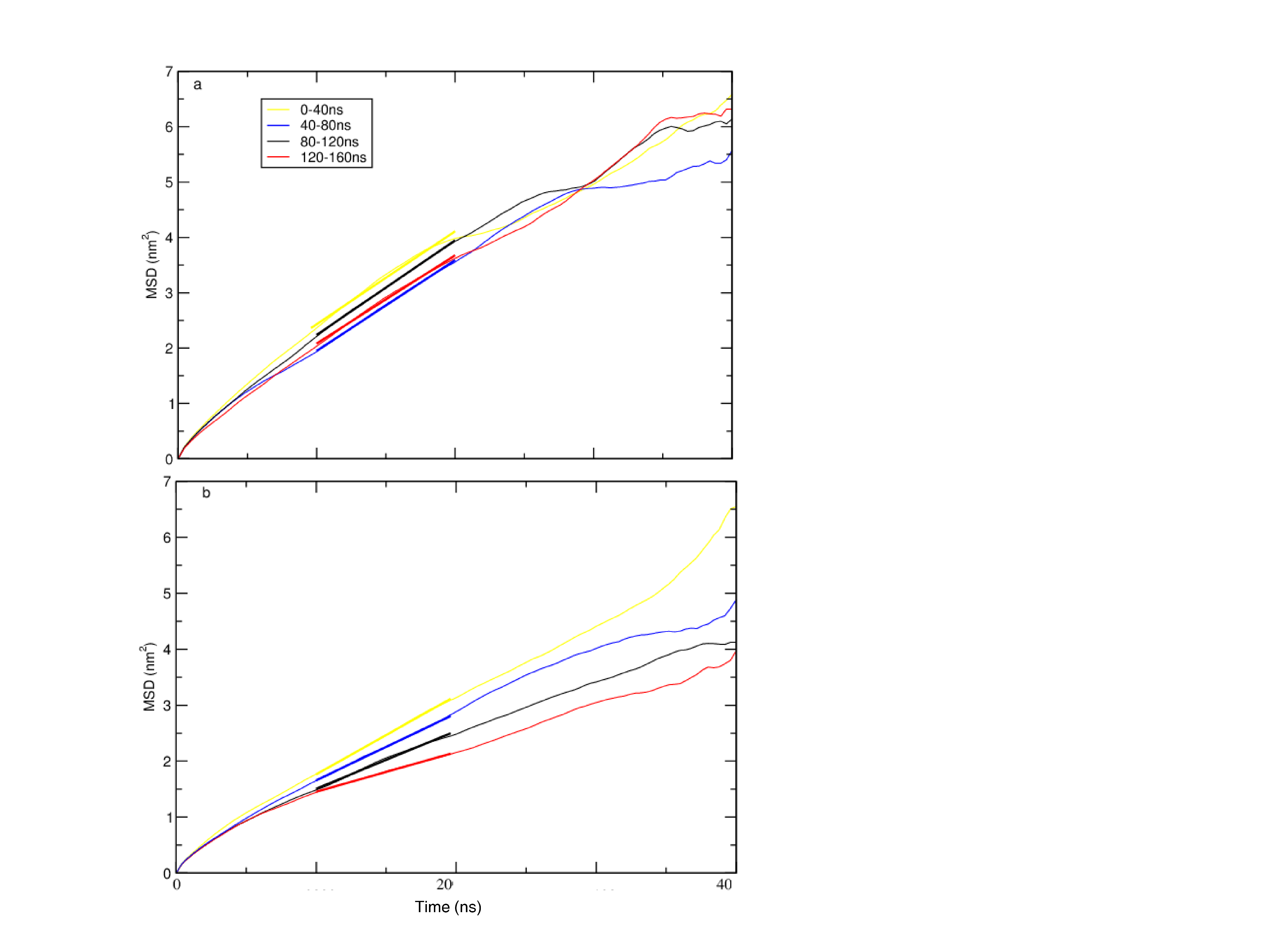

Supplement: Figure S6 — Mean square displacement of H-ras monomers for simulation B1 (a) and B2 (b). The lateral diffusion coefficient was calculated from a linear fit to the portion of the MSD curve highlighted in bold lines and shown in Table 2. (TIF) [file pone.0071018.s006.tif]
