# Supplementary material for: Aggregation of Lipid-Anchored Full-Length H-Ras in Lipid Bilayers: Simulations with the MARTINI Force Field
Source: PLoS One. 2013 Jul 26;8(7):e71018. doi: 10.1371/journal.pone.0071018 (PMC3724741; doi:10.1371/journal.pone.0071018)
Supplement: Table S1 — Additional distance restraints applied in conf2 to keep Ras orientation. (PDF) [file pone.0071018.s007.pdf]

Residue num.	Residue num.	Equilibrium dis.(nm)	Residue num.	Residue num.	Equilibrium dis.(nm)	Remark about the elastic networks
179	180	0.27	174	180	0.96	All backbone beads of residue 170~186 that were within 2.5 nm of each other were explicitly included in the elastic networks, which were used to keep the special orientation of conf2 of H-ras on the PM. The force constant used in this modified elastic network was 500 (kJ mol^{-1} nm^{-2}). Higher values up to 1000 (kJ mol^{-1} nm^{-2}) were also tested but did not make a difference in the result.
	182	0.92		181	1.06	
	184	1.15		182	1.20	
	185	1.16		183	1.47	
	186	1.40		184	1.72	
178	182	1.00		185	1.82	
	183	1.18		186	2.11	
	184	1.44	173	180	1.14	
	185	1.47		181	1.17	
	186	1.72		182	1.25	
177	181	0.97		183	1.54	
	182	1.15		184	1.77	
	183	1.38		185	1.90	
	184	1.61		186	2.21	
	185	1.62	172	180	0.98	
	186	1.88		181	0.93	
176	180	1.04		182	0.98	
	181	1.23		183	1.28	
	182	1.44		184	1.49	
	183	1.67		185	1.62	
	184	1.93		186	1.93	
	185	1.97	171	180	1.18	
	186	2.24		181	1.05	
175	180	1.04		182	1.03	
	181	1.22		183	1.32	
	182	1.39	170	180	1.16	
	183	1.61		181	0.97	
	184	1.88				
	185	1.96				
	186	2.24				
