# Supplementary material for: Aggregation of Lipid-Anchored Full-Length H-Ras in Lipid Bilayers: Simulations with the MARTINI Force Field
Source: PLoS One. 2013 Jul 26;8(7):e71018. doi: 10.1371/journal.pone.0071018 (PMC3724741; doi:10.1371/journal.pone.0071018)
Supplement: Table S2 — Residue pairs for conf1 with high P values (cutoff = 10.0×10 −9 ) over the last two microseconds of the simulations, with residue numbers and single letter codes of the amino acids listed in separate columns. (PDF) [file pone.0071018.s008.pdf]

Resid.#	P values ($\times10^{-9}$)	AA	Resid.#	P values ($\times10^{-9}$)	AA	Resid.#	P values ($\times10^{-9}$)	AA
1 - 42	12.56	M-K	60 - 120	11.68	G-L	120 - 122	11.56	L-A
1 - 43	14.99	M-Q	60 - 121	11.68	G-A	141 - 171	11.35	Y-L
1 - 44	11.77	M-V	61 - 120	10.19	Q-L	141 - 172	10.65	Y-N
12 - 140	10.65	G-P	61 - 121	12.52	Q-A	141 - 173	10.47	Y-P
13 - 140	10.16	G-P	68 - 121	10.77	R-A	147 - 170	10.28	K-K
16 - 140	11.40	K-P	68 - 122	12.12	R-A	148 - 170	10.93	T-K
17 - 140	12.10	S-P	68 - 126	16.88	R-E	148 - 171	16.78	T-L
27 - 149	11.40	H-R	69 - 119	11.59	D-D	148 - 172	16.85	T-N
27 - 150	11.38	H-Q	69 - 120	16.71	D-L	149 - 171	15.71	R-L
27 - 153	11.89	H-E	69 - 121	16.41	D-A	149 - 172	14.34	R-N
28 - 141	11.19	F-Y	69 - 122	13.38	D-A	149 - 173	11.40	R-P
29 - 69	13.78	V-D	70 - 106	12.61	Q-S	150 - 172	11.33	Q-N
30 - 69	11.40	D-D	70 - 107	13.08	Q-D	150 - 173	11.49	Q-P
30 - 141	10.26	D-Y	70 - 118	11.33	Q-C	165 - 167	11.61	Q-K
31 - 69	11.31	E-D	70 - 120	10.68	Q-L	165 - 168	11.17	Q-L
31 - 122	11.80	E-A	70 - 121	10.02	Q-A	166 - 166	11.70	H-H
31 - 141	10.77	E-Y	72 - 105	13.61	M-D	166 - 167	11.40	H-K
32 - 69	11.80	Y-D	72 - 127	11.21	M-S	166 - 168	10.23	H-L
32 - 122	10.16	Y-A	73 - 105	12.17	R-D	167 - 167	10.82	K-K
32 - 140	10.19	Y-P	73 - 106	13.96	R-S	176 - 183	12.77	E-S
32 - 141	10.89	Y-Y	73 - 107	10.19	R-D	176 - 184	13.10	E-4
33 - 68	11.14	D-R	101 - 104	10.91	K-K	177 - 183	20.51	S-S
33 - 126	13.43	D-E	101 - 105	11.24	K-D	177 - 184	20.84	S-4
33 - 134	11.68	D-A	102 - 102	11.28	R-R	177 - 185	16.22	S-L
33 - 139	17.06	D-I	102 - 103	10.98	R-V	178 - 183	16.83	G-S
33 - 140	13.05	D-P	102 - 104	16.67	R-K	178 - 184	16.76	G-4
34 - 68	11.47	P-R	102 - 105	12.00	R-D	178 - 185	13.89	G-L
34 - 70	10.00	P-Q	102 - 127	15.97	R-S	179 - 179	14.41	P-P
34 - 126	10.26	P-E	102 - 128	16.29	R-R	179 - 180	18.51	P-G
34 - 134	13.87	P-A	103 - 103	11.80	V-V	179 - 181	12.33	P-1
34 - 135	12.17	P-R	103 - 104	15.20	V-K	179 - 182	13.82	P-M
34 - 137	12.98	P-Y	103 - 127	15.69	V-S	179 - 183	17.44	P-S
34 - 138	14.48	P-G	103 - 128	11.70	V-R	180 - 180	22.42	G-G
34 - 139	14.76	P-I	104 - 104	11.84	K-K	180 - 181	17.02	G-1
35 - 59	11.63	T-A	104 - 105	11.56	K-D	180 - 182	15.94	G-M
35 - 140	12.05	T-P	104 - 167	11.33	K-K	180 - 183	16.6	G-S
35 - 172	11.68	T-N	105 - 167	12.94	D-K	181 - 181	14.36	1-1
36 - 172	11.40	I-N	105 - 168	10.91	D-L	181 - 182	13.89	1-M
37 - 38	11.56	E-D	106 - 134	10.00	S-A	181 - 183	13.05	1-S
37 - 39	11.31	E-S	106 - 167	13.08	S-K			
38 - 38	11.80	D-D	106 - 168	12.96	S-L			
38 - 39	10.70	D-S	107 - 137	10.33	D-Y			
39 - 39	11.10	S-S	107 - 168	11.00	D-L			
47 - 176	11.82	D-E	107 - 169	12.14	D-R			
48 - 176	11.96	G-E	107 - 170	10.89	D-K			
58 - 121	10.05	T-A	119 - 120	11.07	D-L			
59 - 120	10.68	A-L	119 - 121	11.91	D-A			
59 - 121	11.38	A-A	119 - 122	12.59	D-A			
			120 - 120	11.61	L-L			
			120 - 121	11.38	L-A			
