# Supplementary material for: Aggregation of Lipid-Anchored Full-Length H-Ras in Lipid Bilayers: Simulations with the MARTINI Force Field
Source: PLoS One. 2013 Jul 26;8(7):e71018. doi: 10.1371/journal.pone.0071018 (PMC3724741; doi:10.1371/journal.pone.0071018)
Supplement: Table S3 — Residue pairs for conf2 with high P values (cutoff = 10.0×10 −9 ) over the last two microseconds of the simulations, with residue numbers and single letter codes of the amino acids listed in separate columns. (PDF) [file pone.0071018.s009.pdf]

Resid.#	P values $(\times 10^{-9})$	AA
24 - 148	11.59	I-T
25 - 64	11.11	Q-Y
25 - 65	14.71	Q-S
25 - 98	13.22	Q-E
25 - 127	11.21	Q-S
26 - 63	10.28	N-E
26 - 64	19.95	N-Y
26 - 65	18.70	N-S
26 - 66	13.52	N-A
26 - 98	14.24	N -E
26 - 125	15.41	N-V
26 - 126	12.24	N-E
26 - 127	11.70	N-S
27 - 64	20.44	H-Y
27 - 65	17.97	H-S
27 - 125	13.99	H-V
30 - 120	11.54	D-L
30 - 121	12.26	D-A
35 - 147	12.54	T-K
35 - 148	10.89	T-T
36 - 149	10.33	I-R
44 - 106	10.63	V-S
45 - 106	11.00	V-S
68 - 126	11.70	R-E
69 - 126	10.75	D-E
74 - 121	11.63	T-A
74 - 122	10.61	T-A
75 - 121	10.26	G-A
91 - 148	17.58	E-T
91 - 149	16.27	E-R
92 - 148	12.59	D-T
92 - 149	11.82	D-R
93 - 147	10.14	I-K
93 - 148	14.31	I-T
94 - 148	20.42	H-T
94 - 149	17.58	H-R
94 - 150	15.85	H-Q
95 - 148	11.73	Q-T
95 - 149	11.49	Q-R
101 - 147	10.79	K-K
102 - 128	11.07	R-R
103 - 128	11.66	V-R
105 - 121	10.23	D-A
105 - 146	18.97	D-A
105 - 147	17.24	D-K
106 - 123	10.09	S-R
106 - 146	15.55	S-A
106 - 147	17.97	S-K
106 - 148	13.82	S-T
106 - 149	13.05	S-R
107 - 146	10.00	D-A
107 - 147	15.38	D-K
107 - 148	18.58	D-T
107 - 149	10.37	D-R
108 - 146	11.03	D-A
108 - 148	10.07	D-T
118 - 118	10.21	C-C
118 - 119	10.00	C-D
119 - 146	10.00	D-A
119 - 147	13.57	D-K
120 - 121	11.56	L-A
120 - 146	11.91	L-A
121 - 121	11.70	A-A
144 - 148	10.12	T-T
145 - 147	11.56	S-K
145 - 148	10.98	S-T
148 - 150	12.42	T-Q
148 - 166	10.44	T-H
149 - 149	14.03	R-R
149 - 150	19.84	R-Q
149 - 165	10.72	R-Q
149 - 166	12.59	R-H
149 - 167	10.70	R-K
150 - 150	16.32	Q-Q
150 - 166	11.94	Q-H
150 - 167	11.72	Q-K
154 - 165	10.00	D-Q
154 - 166	13.31	D-H
176 - 176	12.82	E-E
